# Assessment of Rare Genetic Variants to Identify Candidate Modifier Genes Underlying Neurological Manifestations in Neurofibromatosis 1 Patients

**DOI:** 10.3390/genes13122218

**Published:** 2022-11-26

**Authors:** Jie Tang, Niu Li, Guoqiang Li, Jian Wang, Tingting Yu, Ruen Yao

**Affiliations:** Department of Medical Genetics and Molecular Diagnostic Laboratory, Shanghai Children’s Medical Center, School of Medicine, Shanghai Jiao Tong University, Shanghai 200127, China

**Keywords:** NF1, rare variants, modifier gene, nervous system, genetic diagnosis

## Abstract

Neurological phenotypes such as intellectual disability occur in almost half of patients with neurofibromatosis 1 (NF1). Current genotype–phenotype studies have failed to reveal the mechanism underlying this clinical variability. Despite the presence of pathogenic variants of NF1, modifier genes likely determine the occurrence and severity of neurological phenotypes. Exome sequencing data were used to identify genetic variants in 13 NF1 patients and 457 healthy controls, and this information was used to identify candidate modifier genes underlying neurological phenotypes based on an optimal sequence kernel association test. Thirty-six genes were identified as significant modifying factors in patients with neurological phenotypes and all are highly expressed in the nervous system. A review of the literature confirmed that 19 genes including *CUL7*, *DPH1*, and *BCO1* are clearly associated with the alteration of neurological functioning and development. Our study revealed the enrichment of rare variants of 19 genes closely related to neurological development and functioning in NF1 patients with neurological phenotypes, indicating possible modifier genes and variants affecting neurodevelopment. Further studies on rare genetic variants of candidate modifier genes may help explain the clinical heterogeneity of NF1.

## 1. Introduction

Neurofibromatosis type 1 (NF1; MIM#162200) is one of the most common inherited neurogenic disorders, with a worldwide birth incidence of 1 in 2500 [1]. Dominant loss-of-function mutations involving NF1, located on chromosome 17q11.2, are responsible for NF1. Clinical characteristics of NF1 develop with age and include café-au-lait spots, axillary and inguinal freckling, cutaneous and subcutaneous neurofibromas, and Lisch nodules. Other less common features include plexiform neurofibromas, learning disabilities, and musculoskeletal features such as osteopenia, dysplasia of the long bones, and scoliosis [2]. Pharmacological therapy for plexiform neurofibromas can control the development of malignant peripheral nerve sheath tumors, which are thought to be the most serious disease feature. Neurological manifestations represent one of the most frequent findings in NF1 patients and tend to be of the greatest consequence to patients, since there is no treatment that effectively improves quality of life. Learning and language difficulties, together with inattention, hyperactivity, or impulsivity, are recorded in 30% to 60% of cases [3]. Other neurological manifestations include headache, cognitive impairment, motor deficit, and epilepsy [4]. Although several studies have revealed genotype–phenotype correlations for specific NF1 mutations, the clinical expression of NF1 is highly variable and unpredictable, even among patients with the same mutation [5].

Currently, no evidence directly shows whether neurological abnormalities in children with NF1 including learning impairment, intellectual impairment, and autism are related to a specific mutation. Further, no correlation between the disease onset and neurological symptom severity has been shown, even in patients harboring the same genetic variants [6]. Variations in the mutant allele of NF1 itself cannot fully explain disease variability, indicating that other factors, such as modifier genes, environmental factors, second hit somatic events in the *NF1* gene, and epigenetic modification likely underly the phenotypic variability of NF1. Neurological symptoms are likely to be affected by environmental factors. However, the incidence of neurological symptoms in children with NF1 is much higher than that which would be predicted due the impact of environmental factors [7]. Evidence from twin studies suggests that variability may be caused by unidentified modifier genes [8,9]. Genetic modifiers, defined as genetic variants that can modify the phenotypic outcome of a primary disease-causing variant, have been shown to affect phenotypes of some Mendelian disorders [10,11]. Thus, the existence of rare variants of potential modifier genes and their impact on the phenotypic variability of neurological development in NF1 patients warrants further investigation.

In this study, we evaluated the collective effects of rare variants on the modification of neurodevelopmental phenotypes of NF1. For this purpose, information regarding the presence of variants among 13 patients with NF1 who had various degrees of neurological involvement was analyzed. Subsequently, we performed an optimal sequence kernel association test (SKAT-O) to identify genetic modifiers of neurological phenotypes. Our preliminary study provides new insights into possible modifier genes and variants in rare diseases. The list of novel candidate modifier genes and disease pathways may help researchers better understand the cause of phenotypic variability in patients with NF1.

## 2. Materials and Methods

### 2.1. Patient Enrollment

Patients previously diagnosed with NF1 at the Department of Medical Genetics of Shanghai Children’s Medical Center, Shanghai Jiaotong University School of Medicine (Shanghai, China) were included in this study. Ethical approval for this study was obtained from the ethics committee of Shanghai Children’s Medical Center, Shanghai Jiaotong University School of Medicine (protocol code SCMCIRB-K2020060-1; date of approval: 16 June 2020). Written informed consent was obtained from each proband’s parents. Patients’ medical charts were reviewed to identify information related to neurological involvement including learning disabilities, behavioral problems, autism spectrum disorder, attention deficit hyperactivity disorder, seizures, and sleep disturbance. Genomic data of healthy adults without any neurological involvement evaluated by physicians were selected as control.

### 2.2. Sample Preparation

Peripheral blood samples were collected from patients and their parents. The SureSelect Human All Exon V6 enrichment kit (Agilent, Santa Clara, CA, USA) was used to prepare a sequencing library. The experimental process included the enzymatic digestion of DNA fragments, library hybridization, and library amplification and purification. The Hiseq2500 sequencing platform (Illumina, Inc., San Diego, CA, USA) was used for high-throughput sequencing. FastQC version 0.11.9 (Babraham Research Institute, Cambridge, UK) and Fastp version.0.20.1 (Visible Genetics, Inc., Toronto, ON, Canada) were used for data quality control and to remove the adaptor sequence. Speedseq version 0.1.2 (Ira Hall Lab, St. Louis, MO, USA) was used to perform sequence alignment to reference genome. Further, bamdst (version 1.0.9) and mosdepth (version 0.3.1) were used to count sequencing indexes of BAM files after alignment including mapping rate, polymerase chain reaction duplication rate, average sequencing depth, and coverage rate. Genome Analysis Toolkit version 4.2.0.0 (Broad Institute, Cambridge, MA, USA) was used to detect variants in the BAM file passing a quality-control test that was performed in accordance with the best-practice guideline [12]. We began by calling variants per sample to produce a file in GVCF format. Next, we consolidated GVCFs from multiple samples within a GenomicsDB database. We then performed joint genotyping. Finally, VQSR filtering was applied to produce the final multi-sample call set with the desired balance of precision and sensitivity. Copy number variants (CNVs) were identified using the open-source CNVkit software (version 0.9.8), which is a tool kit that can infer and visualize copy numbers from targeted DNA sequencing data [13].

**Figure 1:**
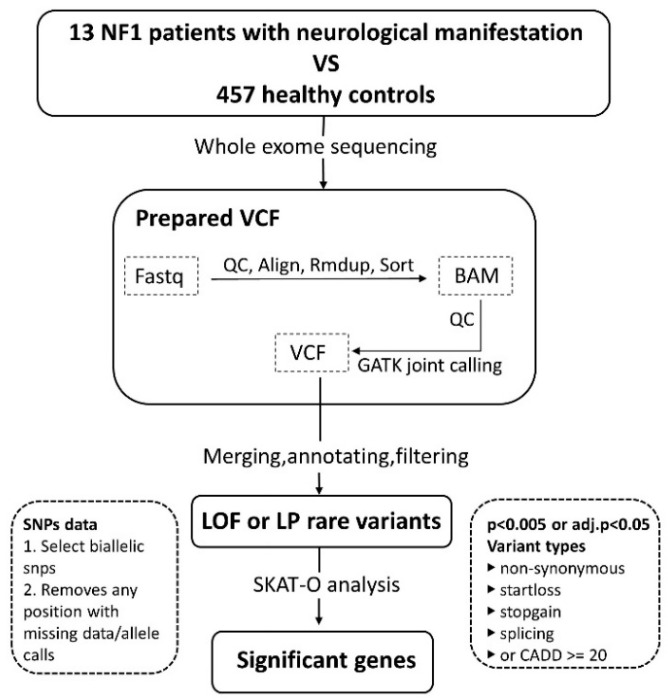
Workflow of rare-variant association analysis. In this study, we recruited 13 NF1 patients and 457 healthy controls and performed whole-exome sequencing for subsequent analysis. VCF files were generated following the best-practice guideline of GATK and rare-variant association analysis was based on the SKAT-O method using LOF/LP variants.

All VCF files of NF1 cases and controls were merged using bcftools (v.1.12). Variants were annotated using ANNOVAR (version 8 June 2020) with sequence ontologies, MAFs (minor allele frequencies) from the Genome Aggregation Database (gnomAD, v.2.1.1), and in silico prediction scores from Combined Annotation Dependent Depletion (CADD), (v.1.3). Rare variants with an MAF <0.01 in gnomAD were included in subsequent analyses. Based on annotations, we collected two groups of variants according to their variation type and possibility of pathogenicity, as follows: (1) putative loss-of-function (LOF) variants (including nonsynonymous, stop-gain, start-loss, and splicing) and (2) likely pathogenic (LP) variants (including missense variants with a CADD phred ≥20) (Figure 1).

### 2.3. Statistics

The SKAT-O was developed by estimating the correlation parameter in the kernel matrix, producing a value equal to the estimated weight of the linear combination of the burden and SKAT test statistics that maximizes power [14]. Thus, we were able to flexibly apply the test in the following two common analysis scenarios: (1) a region with many noncausal variants or causal variants having different directions of association and (2) a region with a high proportion of causal variants with an association in the same direction.

The SKAT-O test was implemented using the R package “SKAT” method of Seunggeun Lee. This method aggregates individual SNP score statistics within an SNP set and efficiently computes SNP set *p*-values. SKAT-O statistics were calculated using putative LOF and LP variants. Only genes with *p*-values < 0.005 were reported (Figure 1).

Follow-up analysis was performed on SKAT-O results with a detected signal using Firth logistic regression. The brglm2 R package was used to fit the regression model and apply a mean bias reduction accounting for the low variant-positive counts. Genes with total rare variant counts <5 between the case and control cohorts were excluded from the analysis.

### 2.4. Pathway Analysis

GO, KEGG, and Reactome pathway associations between genes flagged as significant via SKAT-O statistics were performed using the cluster Profiler R package [15,16,17]. For the gene-set enrichment analysis, all genes listed in the database were used as background. The main goal of the pathway analysis was to group genes of interest into pathways and explore the possibility that these pathways may be involved in the modulation of neurological phenotypes of NF1. An additional search of the PubMed database was performed to determine functional data of identified genes. Gene interaction networks were plotted using STRING and GeneMANIA [18,19].

## 3. Results

### 3.1. Patient Characteristics

Thirteen patients diagnosed with NF1 using NIH diagnostic criteria were included in the analysis. The average age of the patients was 7 years and 2 months, and the average age at initial diagnosis was 4 years and 9 months. Pathogenic variants in or encompassing NF1 were detected through whole-exome sequencing and data analysis. Further validation within a pedigree confirmed that 10 patients carried de novo variants of NF1. Two patients (19654 and 20586) inherited pathogenic NF1 variants from their parents who were also diagnosed with NF1 but without any neurological manifestations. All patients were evaluated within the Department of Developmental Behavioral Pediatrics for neurological manifestations (Table 1). A total of 457 Chinese healthy adults (average age 32 years and 2 months) without any neuropsychiatric symptoms was selected as controls in this study.

### 3.2. Variant Calling and Analysis

GATK yielded an average of 325,476 variants among different samples that passed quality control measures, with a mean value of 1056 rare variants (AF_eas < 0.01) per sample (Supplementary Figure S1). After filtering using two criteria (Figure 1), 439,148 rare LOF variants and 70,658 rare LP variants were considered in the SKAT-O analysis.

Whole-exome sequencing was performed in 13 NF1 patients with neurological manifestation and 457 healthy controls. Rare variants were called using GATK, and filtered according to variant types or CADD phred. Finally, SKAT-O was performed leading to 130 and 127 potential modifier genes, respectively, when applied to the LOF and LP variants. In total, 36 common potential modifier genes (excluding *NF1*) were identified (*p*-value < 0.005) (Supplementary Tables S1 and S2).

### 3.3. Enrichment of Identified Genes

**Figure 2:**
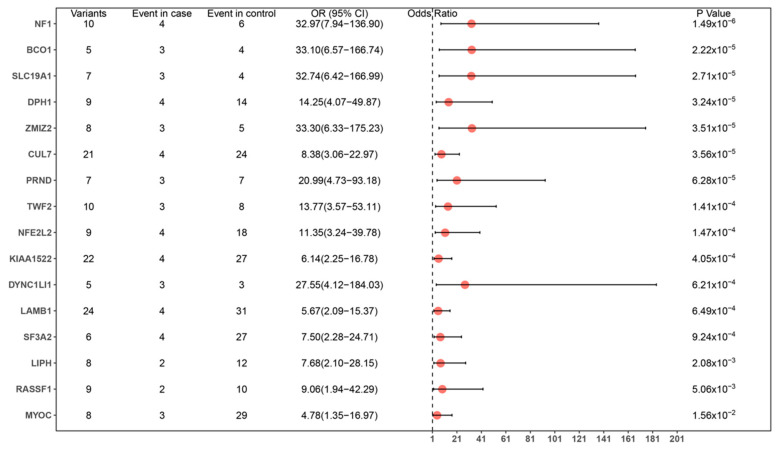
Forest plot showing the odds ratios (ORs), confidence intervals (CIs), and *p*-values for the association between the rare LOF variants of potential modifier genes (unique variants >= 5) in case and control groups. Analyses were performed on SKAT-O results with a detected signal using Firth logistic regression, adjusting for sex, using the brglm2 R package.

**Figure 3:**
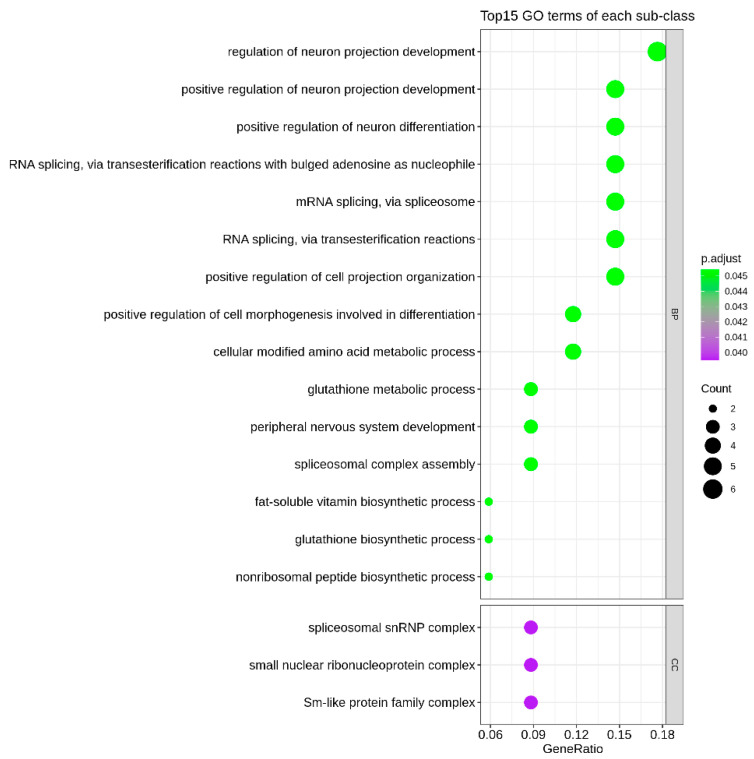
The top 15 enriched GO terms for the identified 37 genes using the clusterProfiler package.

SKAT-O statistics were calculated to determine whether enrichment of rare variants within putative LOF or LP variants was observed. Overall, 36 genes (excluding *NF1*) predicted to significantly modify the neurological phenotype of NF1 were identified in both groups. All identified genes are expressed in the nervous system, and 19 of the 36 have previously been shown to be associated with nervous-system development and functioning (Table 2). The association between rare LOF variants of potential modifier genes (≥5 unique variants) in case and control groups was clearly identified (Figure 2). GO-term analysis of identified potential modifier genes indicated that genes involved in neurological function and development were significantly enriched (Figure 3, Supplementary Table S3). Interaction network evaluation using STRING and GeneMANIA showed close correlations and interactions between potential modifier genes (Supplementary Figure S2).

## 4. Discussion

Incomplete penetrance and phenotypic variability have hindered the clinical diagnosis and study of monogenic Mendelian disorders. Possible explanations for these phenomena are attributed to a combination of genetic, environmental, and lifestyle factors [20], which can make it challenging for genetics professionals to interpret a person’s family medical history and predict the risk of passing a genetic condition to future generations or developing specific phenotypes. At present, the early diagnosis of NF1 is very simple due to genetic testing; however, determining whether and when children will develop clinical phenotypes after early diagnosis requires additional research.

Most individuals with NF1 have normal intelligence, but learning disabilities or behavioral problems occur in 50–80% of patients [21]. Features of autism spectrum disorder occur in up to 30% of children with NF1 [22]. Various other learning and behavioral problems such as visual–spatial skills, memory, executive functioning, and attention problems that persist into adulthood have been described [23]. The genetic burden related to neurological manifestations, such as intellectual disabilities, in patients with 22q11.2 deletion syndrome has been reported [24]. Evidence for the burden of de novo mutations and inherited rare single nucleotide variants in children with sensory processing dysfunction also indicates that genetic background likely influences neurological traits [25]. As for NF1 modifiers, it is reported that heterozygous null mutation of Nmdar I exacerbated the spatial learning phenotype of Nf1 +/− mutant mice [26], and significantly reduced expression levels of neurofibromin isoform I mRNA were found to be correlated with a severe phenotype of NF1 features, including learning disabilities [27]. Hence, interpreting rare variants associated with neurological manifestations in NF1 patients with information generated from whole-exome sequencing data for clinical diagnosis may help researchers identify mechanisms that underlie reduced penetrance and phenotypical variability in these patients.

Modifier genes and relevant variants are assumed to affect mechanisms other than those associated with loss- or gain-of-function mutations, as observed in Mendelian genetic disease genes. These genes may affect disease progression and phenotype severity by influencing multiple processes such as gene expression, transcription, or translation. In this study, we explored the collective effects of rare variants as modifiers of neurological phenotypes of NF1. Since NF1 is a rare progressive disease and neuropsychiatric may occur with the growth of age, we recommend the use of normal healthy controls for association analysis, and genes achieving lower *p*-values are more likely to be neurological modifier genes. A total of 194 rare variants of 36 genes predicted to have damaging effects on phenotypes were enriched in NF1 patients with neurological phenotypes (Supplementary Table S2). All identified genes are expressed in the nervous system, as indicated by various databases including BioGPS (http://biogps.org/, accessed on 15 June 2022) and Genotype-Tissue Expression (GTEx) (https://www.gtexportal.org/, accessed on 15 June 2022). Among the 36 genes, 19 have been associated with nervous system development and functioning in previous studies. Our analysis identified several interesting candidate modifier genes including *CUL7* (*p* = 8.91 × 10^−5^), *DPH1* (*p* = 3.16 × 10^−4^), and *BCO1* (*p* = 3.18 × 10^−4^).

*CUL7* functions as a core component of the Cul7-RING ubiquitin–protein ligase, which mediates ubiquitination and the subsequent degradation of important proteins of the nervous system [28]. Genes encoding the human Eag1 K+ channel have been associated with congenital neurodevelopmental anomalies, and Cul7 is colocalized with Eag1 within synaptic regions of neurons. Cul7 also has been shown to facilitate protein degradation in a disease-associated rEag1 mutant, indicating its possible participation in neurological disorders [29]. *DPH1* encodes an enzyme involved in the biosynthesis of diphthamide, which is involved in intellectual development [30]. Homozygous or compound heterozygous variants of *DPH1* have been reported in patients with developmental delays, unusual skull shape with or without craniosynostosis, sparse hair, and facial dysmorphisms [31]. *DPH1* is within the critical deletion region of Miller–Dieker syndrome (OMIM#247200) and *DPH1* deficiency has been shown to contribute to craniofacial abnormalities [32]. *BCO1* is the enzyme principally responsible for catalyzing the first step in vitamin A biosynthesis. Notably, hypercarotenemia and vitamin A deficiency are associated with mutations in this gene. It also plays a role in regulating miRNAs associated with neuronal differentiation, cell–cell adhesion, and the Wnt signaling pathway [33]. Among candidate modifier genes involved in neurological development and functioning, at least four genes are closely related to Alzheimer’s disease including *FIS1*, *DDX39B*, *PRND*, *GSTM3*. Polymorphisms in these genes are considered risk factors or potential biomarkers for Alzheimer disease [34,35]. Enrichment of rare variants of these genes in NF1 patients with neurological manifestations suggests their potential function in neurodevelopmental aging in addition to neurodegeneration.

The current study had some limitations. The sample size used for assessing rare variants was limited, which is inevitable in studies focusing on rare diseases. To overcome this and increase statistical power from our relatively small cohort, we selected NF1 patients with similar phenotypes (neuropsychiatric symptoms) and screened putative LOF/LP rare variants using SKAT-O, rather than general rare variants. In the presence of positive correlations among the tests, the Bonferroni correction method is conservative and might lack power. For example, an identified disease-causing gene in NF1 patients reached a *p*-value of 5.2 × 10^−5^ in our SKAT-O analysis which was above the Bonferroni-corrected significance threshold. Given the small sample-size of our study, failing to pass the Bonferroni correction threshold could be expected, thus all genes with a *p* < 0.005 in the SKAT-O analysis were moved forward to functional follow-up. Setting the threshold at *p* < 0.005 for SKAT-O analysis could provide significant findings of rare variants, and many studies have already identified interesting signals using a higher *p*-value threshold in SKAT-O analysis [36,37,38]. Further, there was a lack of functional studies and modifier gene validations. Meanwhile, impacts of the accumulation of related rare genetic variants remain unknown. A detailed classification of neurological manifestations in a greater number of NF1 patients may help confirm the importance of identified modifier genes and rare variants. Further, long-term follow-up of diagnosed NF1 patients with and without neurological involvement will benefit in finding genes and variants related to late-onset symptoms.

In conclusion, our study revealed the possible role of rare variants in neurological manifestations of NF1. Analyses based on rare-variant exome sequencing data of NF1 patients and normal controls identified 36 candidate modifier genes. More than half of these genes were found to be associated with normal nervous system development. Further validation of these candidate modifier genes in larger cohorts of NF1 patients is essential. The role of these genes in neurologic abnormalities of NF1 patients requires additional investigations. Interrogating possible modifier genes in genomic data of NF1 patients could provide auxiliary evidence for heterogeneity of the disease. Our study provides new insights into the pathophysiology of NF1 and may improve our ability to provide genetic counseling services to NF1 patients.

## Figures and Tables

**Table 1:** NF1 variants and related neurological involvement in patients.

Patient ID	Gender	Age	Age at Diagnosis	NF1 Variants	Inheritance	NF1-Related Neurological Involvement
14646	Male	10 y	8 y 6 m	c.2125T > C p.Cys709Arg	dn	ADHD, sleep disturbance
14811	Male	12 y 6 m	7 y 9 m	c.3739_3742del p.Phe1247IlefsTer18	dn	Severe learning disabilities, irritability
15098	Female	6 y	4 y 3 m	c.6709C > T p.Arg2237Ter	dn	Autism, severe sleep disturbance
15966	Male	8 y	6 y 9 m	c.5013_5041dup p.Gly1681ValfsTer6	dn	AHDH, autism, psychological problems
16169	Male	14 y	9 y	c.2971_2972del p.Met991AspfsTer29	dn	Migraine headaches, sleep disturbance, mild intellectual disability
17296	Female	9 y	7 y	c.2851-6_2851-3het_del	dn	ADHD, learning problem,
19654	Female	7 y 7 m	4 y 6 m	c.3472G > C p.Asp1158His	Paternal	Language developmental delay, learning disabilities,
19917	Female	7 y	4 y	c.2409 + 1G > C	N/A	autism, ADHD
19928	Male	5 y 3 m	3 y 6 m	c.2180C > A p.Ser727Ter	dn	Epilepsy, language-development delay
20586	Female	4 y 6 m	3 y	c.499_502het_del p.Cys167GlnfsTer10	Maternal	Autism, language-development delay
21178	Male	9 m	6 m	c.1019_1020del p.Ser340CysfsTer12	dn	Epilepsy, cerebral hypoplasia, gross developmental delay
21265	Male	8 y	4 y	c.1019_1020del p.Ser340CysfsTer12	dn	Abnormal brain MRI, intellectual disability
21432	Female	15 m	6 m	c.6974_6977del p.Asp2325ValfsTer49	dn	Epilepsy, gross developmental delay

**Table 2:** The 36 identified candidate modifier genes of neurological involvement in NF1.

Symbol	Name	Associated Monogenic Phenotype (#OMIM)	Expression in Normal Human Nervous Tissue	Association with Neurological System	Pubmed ID
*CUL7*	Cullin 7	3-M syndrome 1	Y	Core component of a Cul7-RING ubiquitin–protein ligase, which mediates ubiquitination and consequent degradation of important proteins of nervous system	21572988
*DUSP26*	Dual specificity phosphatase 26	None	Y	A member of the tyrosine phosphatase family of proteins, which regulates neuronal proliferation	28701747
*OR1L8*	Olfactory receptor family 1 subfamily L member 8	None	Y	Olfactory receptors interact with odorant molecules in the nose, to initiate a neuronal response that triggers the perception of a smell	14983052
*HIGD1C*	HIG1 hypoxia-inducible domain family member 1C	None	Y	None	
*SLC19A1*	Solute carrier family 19 member 1	?Megaloblastic anemia, folate-responsive	Y	A transporter of folate, which may play a role in the pathogenesis of pediatric brain tumors	18406541
*DPH1*	Diphthamide biosynthesis 1	Developmental delay with short stature, dysmorphic facial features, and sparse hair	Y	An enzyme involved in the biosynthesis of diphthamide, which is involved in intellectual development	25558065, 26220823
*BCO1*	β-carotene oxygenase 1	?Hypercarotenemia and vitamin A deficiency, autosomal dominant	Y	Plays a role in regulating miRNAs associated with neuronal differentiation, cell–cell adhesion, and the Wnt signaling pathway	31048207
*ZMIZ2*	Zinc finger MIZ-type containing 2	None	Y	None	
*GSTM3*	Glutathione S-transferase Mu 3	None	Y	Gene variations in GSTM3 are a risk factor for Alzheimer’s disease	18423940, 17904251
*PRND*	Prion-like protein doppel	None	Y	Polymorphisms within the PRND are associated with the Alzheimer’s disease and Creutzfeldt–Jakob disease	11702213, 14745079, 19422537
*TWF2*	Twinfilin actin-binding protein 2	None	Y	None	
*RASSF1*	Ras association domain family member 1	None	Y	The inactivation of RASSF1A may be an important step in the tumorigenesis of the central nervous system	14586413, 19165202, 17899687
*ASCC1*	Activating signal cointegrator 1 complex subunit 1	Barrett esophagus/esophageal adenocarcinoma Spinal muscular atrophy with congenital bone fractures 2	Y	May play a role in the development of neuromuscular junction	28218388, 34204919
*NFE2L2*	NFE2-like BZIP transcription factor 2	Immunodeficiency, developmental delay, and hypohomocysteinemia	Y	NFE2L2 promotes neuronal cell differentiation and controls neuroinflammation. NFE2L2 deregulation has been linked to neurodegenerative diseases	19573594, 32545924, 29969760, 26541884
*PARP6*	Poly(ADP-ribose) polymerase family member 6	None	Y	A regulator of dendrite morphogenesis in hippocampal neurons	34067418, 26725726
*LIPH*	Lipase H	Hypotrichosis 7 Woolly hair, autosomal recessive 2 with or without hypotrichosis	Y	None	
*SF3A2*	Splicing factor 3a subunit 2	None	Y	None	
*KIAA1522*	KIAA1522	None	Y	None	
*LAMB1*	Laminin subunit β 1	Lissencephaly 5	Y	Involved in the organization of the laminar architecture of cerebral cortex	23472759, 21370991
*DYNC1LI1*	Dynein cytoplasmic 1 light intermediate chain 1	None	Y	DYNC1I1 deficiency causes neuronal atrophy in primary hippocampal neurons	27510948
*MYOC*	Myocilin	Glaucoma 1A, primary open angle	Y	Mediates myelination in the peripheral nervous system and plays a role in neurite outgrowth	23897819, 12799138
*U2SURP*	U2 SnRNP-associated SURP domain-containing	None	Y	None	
*DDX39B*	DExD-box helicase 39B	None	Y	Association of DDX39B polymorphisms with Alzheimer’s disease	18715507
*ZMAT2*	Zinc finger matrin-type 2	None	Y	None	
*GFI1*	Growth factor independent 1 transcriptional repressor	None	Y	Important for neuroendocrine differentiation and regulation of neurite outgrowth	15466176, 19026687
*SNAPC5*	Small nuclear RNA-activating complex polypeptide 5	None	Y	None	
*RBPMS*	RNA-binding protein, MRNA-processing factor	None	Y	A selective marker of ganglion cells in the mammalian retina	24318667
*DBF4*	DBF4 zinc finger	None	Y	None	
*DMAC2L*	Distal membrane arm assembly component 2 like	None	Y	None	
*HIST1H3E*	H3 clustered histone 6	None	Y	None	
*FIS1*	Fission, mitochondrial 1	None	Y	Potential biomarkers for Alzheimer’s disease	22340708
*RIT1*	Ras-like without CAAX 1	Noonan syndrome 8	Y	RIT1 cooperates with nerve growth factor to promote neuronal development and regeneration	15632082 12668729
*TRAPPC5*	Trafficking protein particle complex subunit 5	None	Y	None	
*CELF3*	CUGBP Elav-like family member 3	None	Y	None	
*LRRC3C*	Leucine-rich repeat-containing 3C	None	Y	None	
*GCLM*	Glutamate–cysteine ligase-modifier subunit	(Myocardial infarction, susceptibility to)	Y	None	

## Data Availability

The data presented here are available on request from the corresponding author. The data are not publicly available due to privacy and ethical issues.

## Supplementary Materials

The following supporting information can be downloaded at: https://www.mdpi.com/article/10.3390/genes13122218/s1, Figure S1: Summary of rare variants that passed the quality control criteria detected by GATK among 470 samples; Figure S2: Gene interaction networks constructed by STRING and GeneMANIA; Table S1: 37 significant genes using SKAT-O test; Table S2: Rare variants of 37 significant genes used in SKAT-O test; Table S3: Top15 GO terms enriched in 37 significant genes.
