# Supplementary material for: Assessment of Rare Genetic Variants to Identify Candidate Modifier Genes Underlying Neurological Manifestations in Neurofibromatosis 1 Patients
Source: Genes (Basel). 2022 Nov 26;13(12):2218. doi: 10.3390/genes13122218 (PMC9778305; doi:10.3390/genes13122218)
Supplement: Supplementary file 1 [file genes-13-02218-s001.zip › Supplemental Information.pdf]

Supplemental Information

Supplemental Figure S1.

Summary of rare variants that passed the quality control criteria detected by GATK among 470 samples.

Supplementary Figure S2.

(a) Thirty-seven genes determined to be significantly associated with an interaction network using STRING. The PPI network was constructed based on the following seven parameters: text mining, experiments, databases, co-expression, neighborhood, gene fusion, and co-occurrence. The K-means method was then applied for clustering, resulting in five clusters indicated with different colors. (b) Thirty-seven genes determined to be significantly associated with an interaction network using GeneMANIA. The PPI network was constructed based on the following four factors: co-expression, co-localization, physical interactions, and genetic interactions. Line thickness indicates the strength of data support.
