# Supplementary figures and images for: Assessment of Rare Genetic Variants to Identify Candidate Modifier Genes Underlying Neurological Manifestations in Neurofibromatosis 1 Patients
Source: Genes (Basel). 2022 Nov 26;13(12):2218. doi: 10.3390/genes13122218 (PMC9778305; doi:10.3390/genes13122218)

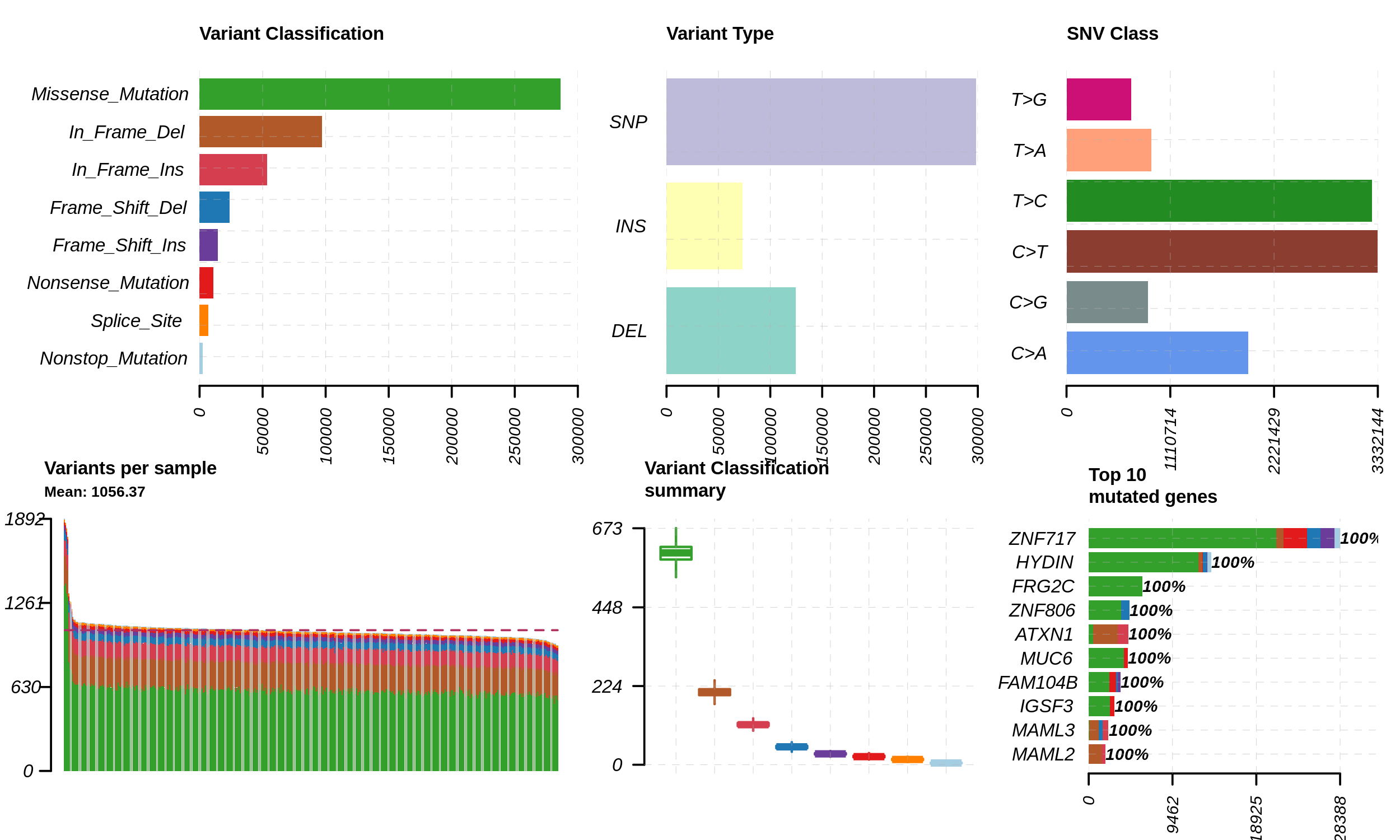

Supplement: Supplementary file 1 [file genes-13-02218-s001.zip › Figure S1.png]

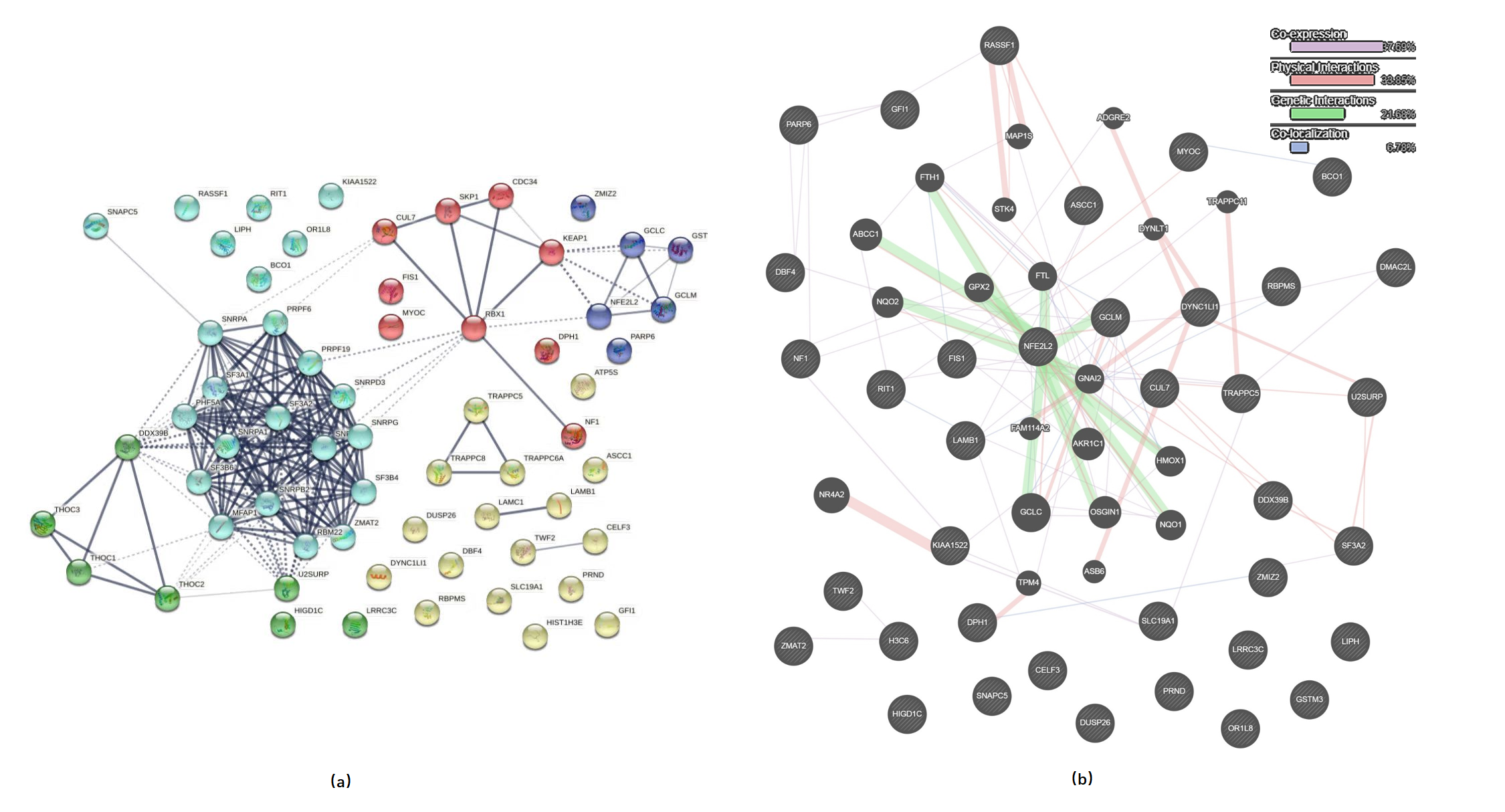

Supplement: Supplementary file 1 [file genes-13-02218-s001.zip › Figure S2.png]
